# Adaptive Momentum Langevin Dynamics for Efficient Posterior Sampling

**DOI:** 10.3390/e28080854

**Published:** 2026-08-01

**Authors:** Zhengbo Li, Jian Xu, Dingtao Peng, Shuang Hu

**Affiliations:** 1 School of Mathematics and Statistics, Guizhou University, Guiyang 550025, China; lizhengbo@mtxy.edu.cn; 2 School of General Education, Moutai Institute, Renhuai 564507, China; hu854304661@163.com; 3 School of Mathematics, South China University of Technology, Guangzhou 510640, China; jian.xu@a.riken.jp; 4 Guizhou Provincial Laboratory of Big Data, Guizhou University, Guiyang 550025, China

**Keywords:** adaptive Langevin dynamics, underdamped Langevin dynamics, Bayesian inference, posterior sampling, stochastic sampling, score-based priors, inverse problems, image restoration

## Abstract

Sampling from high-dimensional posterior distributions is a central challenge in Bayesian inference and noisy inverse problems. Standard first-order Langevin-based methods often suffer from slow convergence and sensitivity to step-size hyperparameters, particularly in annealed score-based inverse imaging pipelines. We propose Adaptive Momentum Langevin Dynamics (AMLD), a practical stochastic correction kernel that introduces a momentum variable into the annealed posterior sampling framework and equips it with an annealing-aware momentum retention schedule. The method is fully compatible with the SNIPS framework and retains its coordinate-wise adaptive step structure, acting as a lightweight drop-in replacement for the conventional first-order Langevin correction step. Extensive experiments on three representative image inverse problems—Gaussian deblurring, inpainting, and 4× super-resolution—demonstrate that AMLD consistently achieves strong PSNR and LPIPS performance, with competitive FID in most settings, compared to three state-of-the-art baselines (DDRM, DPS, SNIPS) under both nearly noiseless and noisy measurement conditions, while reaching target reconstruction quality using fewer sampling iterations. The proposed momentum-based sampler provides empirically improved exploration and robustness across evolving posterior landscapes, offering a practical and computationally efficient alternative to first-order annealed Langevin samplers in high-dimensional Bayesian inverse problems.

## 1. Introduction

Sampling from high-dimensional posterior distributions is a central problem in Bayesian inference and inverse problems [1,2]. Langevin-based methods provide a principled framework, but often suffer from slow convergence and sensitivity to hyperparameters [3,4,5]. Recent approaches such as SNIPS [6] integrate learned score-based priors but rely on basic annealed Langevin dynamics with fixed friction, limiting exploration in multimodal landscapes.

Concurrently, adaptive optimizers like Adam [7] have revolutionized non-convex optimization through gradient momentum. Our key insight is that these principles can be translated to overcome exploration bottlenecks in posterior sampling.

Our Contributions:

- We introduce an annealing-aware momentum retention schedule that adapts momentum persistence to the evolving posterior geometry across noise levels, improving both early-stage stability and late-stage convergence.
- We design a momentum-based correction kernel that is fully compatible with the SNIPS framework and preserves its coordinate-wise adaptive step mechanism, serving as a lightweight drop-in replacement for the standard first-order Langevin update.
- We empirically demonstrate improved reconstruction quality and sampling efficiency on three benchmark image inverse problems under both noiseless and noisy conditions, and provide an ablation study quantifying the contribution of each component.

Unlike Adam-type preconditioning, which rescales gradients in overdamped dynamics, our approach introduces a momentum retention variable within the annealed sampling framework. This leads to a different effect: instead of rescaling gradient magnitudes per coordinate, it adds directional persistence to the correction trajectory, thereby accelerating exploration and reducing random-walk behavior. We emphasize that AMLD is designed as a practical stochastic sampler rather than an exact MCMC method with strict theoretical guarantees. Our method is fully compatible with existing inference pipelines such as SNIPS, where it can replace the standard Langevin sampler without modifying other components.

## 2. Related Work

Stochastic Gradient MCMC. SGLD [8] introduced stochastic gradient Langevin dynamics for scalable Bayesian learning, enabling posterior inference with mini-batch gradients. While theoretically well-founded [9], SGLD suffers from slow mixing in high-dimensional or multimodal settings. SGHMC [10] introduced momentum to improve exploration, drawing on Hamiltonian Monte Carlo. pSGLD [5] further proposed using gradient second-moment statistics to precondition the gradient in overdamped dynamics, effectively rescaling the step size per dimension. A key distinction from our method is that pSGLD performs coordinate-wise gradient preconditioning in the overdamped setting, whereas AMLD operates in the underdamped regime and adapts momentum retention at the annealing level rather than per dimension or per step. A complete characterization of valid SG-MCMC update rules is provided in [9], offering a unified perspective on the design space of stochastic gradient samplers. Adaptive MCMC [3] provides a general theoretical framework for adapting proposal distributions while preserving the target stationary distribution asymptotically.

Score-Based Generative Models and Inverse Problems. Score-based generative models [11,12] learn the gradient of the log data density, enabling high-quality image synthesis via Langevin dynamics or stochastic differential equations. SNIPS [6] integrates these learned score functions as priors within an annealed Langevin sampling framework for solving noisy inverse problems, but relies on a fixed scalar friction coefficient throughout. DDRM [13] exploits the singular value decomposition of linear degradation operators to perform efficient diffusion-based restoration, but is restricted to linear measurement models. DPS [14] extends diffusion posterior sampling to general noisy and nonlinear inverse problems via an approximate likelihood score. A comprehensive survey [15] provides a systematic treatment of these approaches, highlighting trade-offs between computational efficiency, measurement consistency, and sample diversity.

Langevin Dynamics and Posterior Sampling. Underdamped Langevin dynamics introduces an auxiliary momentum variable that enables more persistent state-space exploration than purely first-order overdamped dynamics [1]. The connection between Langevin-based dynamics and Bayesian posterior sampling is thoroughly discussed in [1,2], providing the theoretical motivation for momentum-based samplers in score-based inverse problems. AMLD adopts this momentum perspective within the SNIPS annealed sampling framework using a practical finite-step update that does not modify the coordinate-wise step mechanism inherited from SNIPS.

Positioning of AMLD. The proposed method builds on two lines of research: it adopts the momentum-based underdamped perspective of SGHMC [10] to alleviate random-walk behavior, and it introduces an annealing-aware retention schedule tailored to the coarse-to-fine structure of score-based inverse problem samplers such as SNIPS [6]. Unlike pSGLD [5], which performs coordinate-wise preconditioning in the overdamped setting, AMLD operates in the underdamped regime and adapts at the annealing level rather than per step or per dimension. Relative to fixed-momentum underdamped samplers, the annealing-aware schedule provides a lightweight stability refinement that matches the changing posterior geometry across noise levels.

## 3. Method

We present Adaptive Momentum Langevin Dynamics (AMLD) in a step-by-step manner. We first formulate the Bayesian linear inverse problem and define the annealed posterior target used in score-based inverse imaging. We then introduce reference continuous-time underdamped Langevin dynamics that motivates the use of momentum for posterior exploration. Based on this formulation, we develop an annealing-aware momentum schedule that adapts to the changing posterior geometry across noise levels, and derive the discrete update used in practice. Finally, we summarize the complete AMLD algorithm and clarify its methodological role as an efficient posterior correction kernel. Importantly, AMLD does not redefine the posterior target itself; rather, it improves the finite-step stochastic dynamics used to explore that target across annealing levels. AMLD is fully compatible with the SNIPS framework and retains its coordinate-wise adaptive step structure.

### 3.1. Problem Setup

We consider a standard linear inverse problem in imaging. Let $x\in R^{d}$ denote the unknown clean image and let $y\in R^{m}$ denote the observed degraded measurement. The forward observation model is

(1) y=Hx+n,

where $H\in R^{m\times d}$ is a known linear degradation operator and $n\in R^{m}$ denotes additive measurement noise.

Direct inversion of Equation (1) is generally ill-posed: *H* may be ill-conditioned or rank-deficient, and the observation is corrupted by noise. Consequently, multiple clean images may explain the same degraded measurement. Instead of seeking a single point estimate, we adopt a Bayesian formulation and infer the posterior distribution of the unknown image given the observation.

By Bayes’ rule,

(2) p(x∣y)∝p(y∣x)p(x),

where p(y∣x) is the likelihood induced by the forward model and p(x) is the prior distribution of natural images. For Gaussian measurement noise with standard deviation $\sigma_{n}$,

(3) $$p\left(y|x\right)\propto \exp\;\left(-\frac{{∥Hx-y∥}_{2}^{2}}{2\sigma_{n}^{2}}\right).$$

In score-based posterior sampling, rather than sampling directly from the final posterior in one step, one introduces a sequence of noise-conditioned intermediate targets indexed by annealing noise levels $\sigma_{l}$. At annealing level *l*, the corresponding annealed posterior energy is

(4) $$U_{l}\left(x\right)=\frac{{∥Hx-y∥}_{2}^{2}}{2\sigma_{n}^{2}}-\log p_{\sigma_{l}}(x),$$

where $\sigma_{l}$ is the annealing noise level and $p_{\sigma_{l}}(x)$ denotes the Gaussian-smoothed prior distribution associated with the noise-conditioned score model. The annealing mechanism acts through the prior term, while the data-consistency term remains fixed by the observation model.

In score-based generative models, the prior score is approximated by a pre-trained network:

(5) $$s_{\theta }(x,\sigma_{l})\approx \nabla_{\;x}\log p_{\sigma_{l}}(x).$$

Taking the gradient of Equation (4) and substituting Equation (5) gives the annealed posterior energy gradient:

(6) $$\nabla U_{l}(x)=\frac{H^{T}(Hx-y)}{\sigma_{n}^{2}}-s_{\theta }(x,\sigma_{l}).$$

The derivation chain from the observation model to the posterior gradient is

$$\underset{\left(1\right)}{\underset{︸}{y=Hx+n}}\overset{\mathrm{Gaussian}\;\mathrm{noise}}{\to }\underset{\left(3\right)}{\underset{︸}{p(y|x)}}\overset{\mathrm{Bayes}}{\to }\underset{\left(2\right)}{\underset{︸}{p(x|y)}}\overset{-\log}{\to }\underset{\left(4\right)}{\underset{︸}{U_{l}(x)}}\overset{\nabla +\mathrm{score}}{\to }\underset{\left(6\right)}{\underset{︸}{\nabla U_{l}(x)}}.$$

The first term in Equation (6) enforces consistency with the observation via the forward model, while the second incorporates the learned image prior. Posterior sampling is therefore used to generate reconstructions that satisfy both the measurement constraint and the learned prior distribution. This is particularly important for ill-posed inverse problems, where multiple plausible solutions naturally arise.

The objective of AMLD is not to alter this posterior target, but to construct an efficient stochastic correction kernel that approximately explores it through a finite annealed refinement sequence.

### 3.2. Notation and Assumptions

The main notation used throughout Section 3 is summarized in Table 1.

We distinguish three representations of the image variable. The symbol x is the latent image in the Bayesian formulation; $x_{t}$ is the continuous-time process used to motivate the algorithm; and $x_{k}^{\left(l\right)}$ is the discrete iterate at inner step *k* and annealing level *l*. All three refer to the same underlying quantity at different levels of abstraction.

We also distinguish two types of noise. The measurement noise n is part of the physical observation model (Equation (1)), whereas $\sigma_{l}$ is an algorithmic annealing parameter that controls the sequence of intermediate sampling targets.

We assume throughout that (i) *H* is known; (ii) the score network provides a reasonable approximation of the prior score at each noise level; (iii) $\nabla U_{l}$ is sufficiently regular for the updates to be well defined; and (iv) all hyperparameters are chosen to maintain numerical stability.

### 3.3. Annealed Posterior Sampling Framework

Score-based inverse problem methods sample from the sequence ${\left\{p_{l}(x)\propto e^{-U_{l}\left(x\right)}\right\}}_{l=1}^{L}$ of annealed posteriors, gradually refining samples from coarse to fine scales and decomposing posterior sampling into a sequence of easier stochastic correction sub-problems.

At each annealing level *l*, starting from a state passed from the previous level, the sampler performs *K* correction steps intended to move the current state toward the annealed posterior before proceeding to the next level. The gradient required at each step is given by Equation (6).

Following the SNIPS framework, each coordinate is associated with an individual step size encoded in the vector $s_{l}$, derived from the singular value decomposition (SVD) of *H* and adapted to each noise level. This coordinate-wise step mechanism is preserved in full by AMLD. While the posterior score $g_{k}^{\left(l\right)}=-\nabla U_{l}(x_{k}^{\left(l\right)})$ is expressed in unified mathematical form, its practical computation follows the SNIPS piecewise coordinate partitioning based on the SVD of *H*: coordinates are partitioned according to whether $\sigma_{l}$ times the corresponding singular value is larger than, smaller than, or zero at the measurement noise level $\sigma_{0}$. This piecewise decomposition is inherited directly from SNIPS and provides numerical efficiency; it does not alter the mathematical definition of the posterior score.

The role of AMLD is to improve the posterior correction step performed at each annealing level. Specifically, AMLD replaces the conventional first-order Langevin correction by a momentum-based alternative designed to improve empirical exploration efficiency and numerical stability. The resulting procedure should be understood as a practical finite-step sampler for the annealed posterior sequence rather than as an exact simulator of a single invariant diffusion.

### 3.4. Reference Continuous-Time Dynamics

To motivate the use of momentum in AMLD, we consider a reference underdamped Langevin dynamics on an extended state space $\left(x_{t},m_{t}\right)$. With a scalar friction coefficient γ>0, the reference dynamics is

(7) $$dx_{t}=m_{t}\;dt,$$

(8) $$dm_{t}=-\gamma m_{t}\;dt-\nabla U_{l}(x_{t})\;dt+\sqrt{2\gamma }\;dW_{t},$$

where $W_{t}$ is a standard Wiener process. The momentum variable is introduced to accelerate state-space exploration and alleviate the slow random-walk behavior of purely first-order overdamped Langevin dynamics.

This continuous-time system serves as the conceptual motivation for AMLD. The friction term in the reference dynamics motivates momentum persistence, while the practical coefficient $\gamma_{l}\in \left[0,1\right]$ acts as a *momentum retention factor* that controls what fraction of the previous momentum is carried to the next step; it plays an analogous but distinct role to the friction coefficient γ in the continuous reference model. To remain compatible with the coordinate-wise step mechanism of SNIPS, the practical discrete algorithm uses a simplified momentum update that does not aim to be an exact discretization of Equations (7) and (8).

### 3.5. Annealing-Aware Momentum Schedule

Across annealing levels, the posterior landscape changes substantially: at high noise levels the energy surface is broad and requires careful exploration to avoid instability, while at low noise levels the landscape sharpens and larger momentum accelerates convergence toward fine posterior modes. A fixed momentum retention factor cannot adapt to this evolving geometry. AMLD therefore introduces an annealing-aware schedule that increases the momentum retention factor progressively across noise levels.

Let $\gamma_{\min}$ and $\gamma_{\max}$ be the minimum and maximum momentum retention factors, and let r∈(0,1] be the warmup ratio. At annealing level *l* (l=1,…,L), the retention factor is

(9) $$\gamma_{l}=\gamma_{\min}+\left(\gamma_{\max}-\gamma_{\min}\right)\min\;\left(1,\;\frac{(l-1)/L}{r}\right).$$

During the warmup phase, the coefficient gradually increases from $\gamma_{\min}$ toward $\gamma_{\max}$, reaching $\gamma_{\max}$ shortly after the rL-th annealing level. For the remaining levels it stays fixed at $\gamma_{\max}$. Within each annealing level, $\gamma_{l}$ remains constant across all *K* inner correction steps.

This schedule assigns stronger attenuation ($\gamma_{l}$ near $\gamma_{\min}$) in early high-noise stages to prevent momentum from accumulating over rough energy surfaces, and allows larger retention ($\gamma_{l}=\gamma_{\max}$) in later low-noise stages to accelerate convergence.

### 3.6. Discrete Sampling Updates

We describe the discrete AMLD update used in practice. All operations use the coordinate-wise step vector $s_{l}$ to maintain full compatibility with SNIPS. Let $x_{k}^{\left(l\right)}$ denote the position at inner step *k* and annealing level *l*, and let $g_{k}^{\left(l\right)}=-\nabla U_{l}(x_{k}^{\left(l\right)})$ denote the corresponding posterior score computed via Equation (6).

A single auxiliary momentum vector m is maintained throughout the entire sampling procedure; it is initialized to zero once before the annealing schedule begins and *carried continuously across all annealing levels* without being reset between levels.

The momentum update at each inner step is

(10) $$m\;\leftarrow \;\gamma_{l}\;m+s_{l}⊙g_{k}^{\left(l\right)},$$

where ⊙ denotes element-wise multiplication. The first term retains a fraction $\gamma_{l}$ of the accumulated momentum from the previous step, providing directional persistence. The second term incorporates the current posterior score scaled by the coordinate-wise step size, directing momentum toward higher-probability regions of the annealed posterior.

The position update is

(11) $$x_{k+1}^{\left(l\right)}=x_{k}^{\left(l\right)}+m+\sqrt{2s_{l}}⊙\xi_{k},\;\xi_{k}∼N\left(0,I\right).$$

The position is advanced by the accumulated momentum and a stochastic exploration term whose variance is controlled coordinate-wise by $s_{l}$. This differs from the classical underdamped Langevin discretization, where noise typically enters the momentum equation, and is adopted here as a practical SNIPS-compatible design choice rather than an exact invariant-preserving integrator. Injecting noise directly into the position update, rather than into the momentum update, prevents stochastic perturbations from accumulating in the momentum across steps.

Because the discretization is finite-step and the retention factor varies across annealing levels, AMLD does not preserve the exact stationary measure of the reference dynamics in Equations (7) and (8). It is therefore used as a practical posterior correction kernel whose effectiveness is assessed empirically.

### 3.7. Complete AMLD Algorithm

Algorithm 1 presents the complete AMLD procedure. Relative to the SNIPS baseline, the only structural changes are: (i) the addition of a persistent momentum vector m initialized once before the annealing schedule; (ii) the computation of the annealing-aware retention factor $\gamma_{l}$ at the start of each noise level; and (iii) a two-line update replacing the single-line first-order Langevin step. All other components—the score network, annealing schedule, and SVD-based coordinate-wise step vector—are inherited unchanged from SNIPS.
**Algorithm 1** Adaptive Momentum Langevin Dynamics (AMLD)**Require:** Observation y, operator *H*, score network $s_{\theta }$, noise levels ${\left\{\sigma_{l}\right\}}_{l=1}^{L}$, step vectors ${\left\{s_{l}\right\}}_{l=1}^{L}$, inner steps *K*, momentum bounds $\gamma_{\min}$, $\gamma_{\max}$, warmup ratio *r*
 1:Initialize $x_{0}^{\left(1\right)}∼N\left(0,\sigma_{1}^{2}I\right)$ 2:Initialize momentum m=0 {initialized once; carried across all levels} 3:**for** l=1 **to** *L* **do** 4:   Compute retention factor (Equation (9)): $\gamma_{l}=\gamma_{\min}+\left(\gamma_{\max}-\gamma_{\min}\right)\min\;\left(1,\;(l-1)/(rL)\right)$ 5:   **for** k=0 **to** K−1 **do** 6:      Compute posterior score (Equation (6)): $g_{k}^{\left(l\right)}=s_{\theta }(x_{k}^{\left(l\right)},\sigma_{l})-H^{T}(Hx_{k}^{\left(l\right)}-y)/\sigma_{n}^{2}$ 7:      Sample $\xi_{k}∼N\left(0,I\right)$ 8:      Update momentum (Equation (10)): $m\leftarrow \gamma_{l}\;m+s_{l}⊙g_{k}^{\left(l\right)}$ 9:      Update position (Equation (11)): $x_{k+1}^{\left(l\right)}=x_{k}^{\left(l\right)}+m+\sqrt{2s_{l}}⊙\xi_{k}$  10:   **end for**  11:   **if** l<L **then**  12:      $x_{0}^{\left(l+1\right)}=x_{K}^{\left(l\right)}$ {m persists; no reset}  13:   **end if**  14:**end for**  15:**return** $x_{K}^{\left(L\right)}$


AMLD should be interpreted as a practical posterior correction kernel within the annealed sampling framework. It does not alter the annealed posterior targets defined by the observation model and the score prior; instead, it improves the empirical efficiency and robustness of the finite-step stochastic dynamics by introducing momentum and an annealing-aware retention schedule. The additional computational overhead is negligible compared with score-network evaluation, making AMLD a lightweight drop-in replacement for the conventional first-order correction kernel.

## 4. Experiments

### 4.1. Experimental Setup

We evaluate Adaptive Momentum Langevin Dynamics (AMLD) on three representative imaging inverse problems: Gaussian deblurring, image inpainting, and 4× super-resolution. In all tasks, the degradation operator *H* is assumed to be known, and the goal is to recover the underlying clean image from a noisy observation. We follow the standard annealed posterior sampling protocol used in score-based inverse problems and use the same pre-trained score prior and the same degradation settings across all compared methods. Relative to the baseline annealed Langevin sampler, the only change is the replacement of the standard first-order correction step by the proposed AMLD correction kernel.

To regularize these ill-conditioned inverse problems, we employ a pre-trained score network trained on the CelebA dataset (https://mmlab.ie.cuhk.edu.hk/projects/CelebA.html (accessed on 17 July 2026)), which serves as a shared image prior for all methods. For evaluation, we randomly sample 100 validation images from CelebA and compare AMLD against three representative baselines:

- DDRM [13]: A denoising diffusion restoration model that exploits the SVD structure of linear degradation operators for efficient conditional sampling.
- SNIPS [6]: An annealed first-order Langevin posterior sampler built on learned score-based priors.
- DPS [14]: A diffusion posterior sampling method applicable to general noisy and nonlinear inverse problems.

All methods use the same score network to ensure that observed differences are primarily due to the sampling dynamics rather than to changes in the generative prior.

We report PSN (higher is better), LPIPS (lower is better), and FID (lower is better). This combination captures both distortion-oriented and perceptual aspects of reconstruction quality, which is important because posterior samplers for inverse problems often exhibit a distortion–perception trade-off. We consider two observation-noise regimes: a nearly noiseless setting ($\sigma_{0}=0.0002$) and a noisy setting ($\sigma_{0}=0.1$).

#### 4.1.1. Training Details

The score network is trained with the following hyperparameters: CelebA dataset, image size 64×64, 3 channels, random horizontal flip; batch size 128, 500,000 training steps; Adam optimizer [7], learning rate $1\times 10^{-4}$, $\beta_{1}=0.9$, weight decay 0; noise schedule with $\sigma_{\mathrm{begin}}=90$, $\sigma_{\mathrm{end}}=0.01$, geometric distribution, 500 noise classes; InstanceNorm++ normalization, ELU nonlinearity, $n_{gf}=128$, and EMA rate 0.999.

#### 4.1.2. Sampling Parameters

For SNIPS and AMLD, unless otherwise specified, we use sampling batch size 16, step size $\mathrm{lr}=3.3\times 10^{-6}$, and $n_{\mathrm{steps}\;\mathrm{each}}=5$. AMLD introduces a momentum retention factor range $\left[\gamma_{\min},\gamma_{\max}\right]$ and an annealing-level momentum warmup ratio. To illustrate the practical distortion–perception trade-off of AMLD, we report two momentum configurations: a lower-momentum setting with $\gamma_{\min}=0.0$ and $\gamma_{\max}=0.1$, which tends to favor perceptual quality, and a higher-momentum setting with $\gamma_{\min}=0.4$ and $\gamma_{\max}=0.9$, which tends to favor pixel-wise fidelity. These two configurations should not be interpreted as separate model variants; they differ only in the momentum range of the same AMLD sampler, while the score prior, annealing schedule, and observation model remain unchanged. The momentum warmup ratio is set to 0.5 in all cases, meaning the retention factor is linearly ramped from $\gamma_{\min}$ to $\gamma_{\max}$ across the first half of the annealing levels.

#### 4.1.3. Evaluation Protocol

We generate a single reconstruction per image and compute PSNR, LPIPS, and FID over the 100 validation samples. The two AMLD momentum configurations are reported only to illustrate the distortion–perception trade-off and should not be viewed as changing the underlying prior or sampling framework. Unless noted otherwise, all remaining sampling and evaluation settings are kept fixed throughout.

### 4.2. Results on Noiseless Images

Table 2 summarizes performance under the nearly noiseless setting ($\sigma_{0}=0.0002$).

AMLD performs strongly across all three inverse problems in the nearly noiseless regime. For Gaussian deblurring, AMLD achieves the highest PSNR (40.02 dB) and the lowest LPIPS (0.0006), outperforming all baselines on distortion and perceptual similarity, while remaining competitive in FID. For inpainting, AMLD surpasses all competing methods on all three metrics, with clear gains in both fidelity and perceptual quality. For 4× super-resolution, AMLD again yields the best results across PSNR, LPIPS, and FID, including a PSNR gain of more than 2 dB over DDRM.

Overall, these results suggest that AMLD provides a more effective posterior correction process than standard first-order Langevin updates when the signal-to-noise ratio is high. Empirically, the combination of momentum and the annealing-aware schedule leads to sharper reconstructions, fewer oversmoothed regions, and improved measurement-consistent detail recovery.

### 4.3. Results on Noisy Images

Table 3 reports results in the noisy setting ($\sigma_{0}=0.1$).

Under noise, the performance gap between methods narrows, but AMLD remains consistently competitive and often achieves the best overall reconstruction quality. For Gaussian deblurring, AMLD attains the highest PSNR (26.48 dB), slightly exceeding DDRM while maintaining competitive LPIPS and FID. For inpainting, AMLD achieves the best PSNR (20.14 dB) and LPIPS (0.0420), indicating strong robustness to measurement noise. For 4× super-resolution, AMLD obtains the best FID (105.00) and the highest PSNR (22.32 dB), suggesting improved preservation of fine structures in a more challenging posterior landscape.

Taken together, the noisy and nearly noiseless results indicate that AMLD is not limited to a single operating regime. Although no single method dominates every metric on every task, AMLD consistently achieves top or near-top performance across a broad range of conditions. This empirical behavior supports the use of adaptive momentum-based correction dynamics as a practical alternative to standard first-order annealed Langevin updates in score-based inverse problems.

### 4.4. Efficiency Analysis

**Figure 1:**
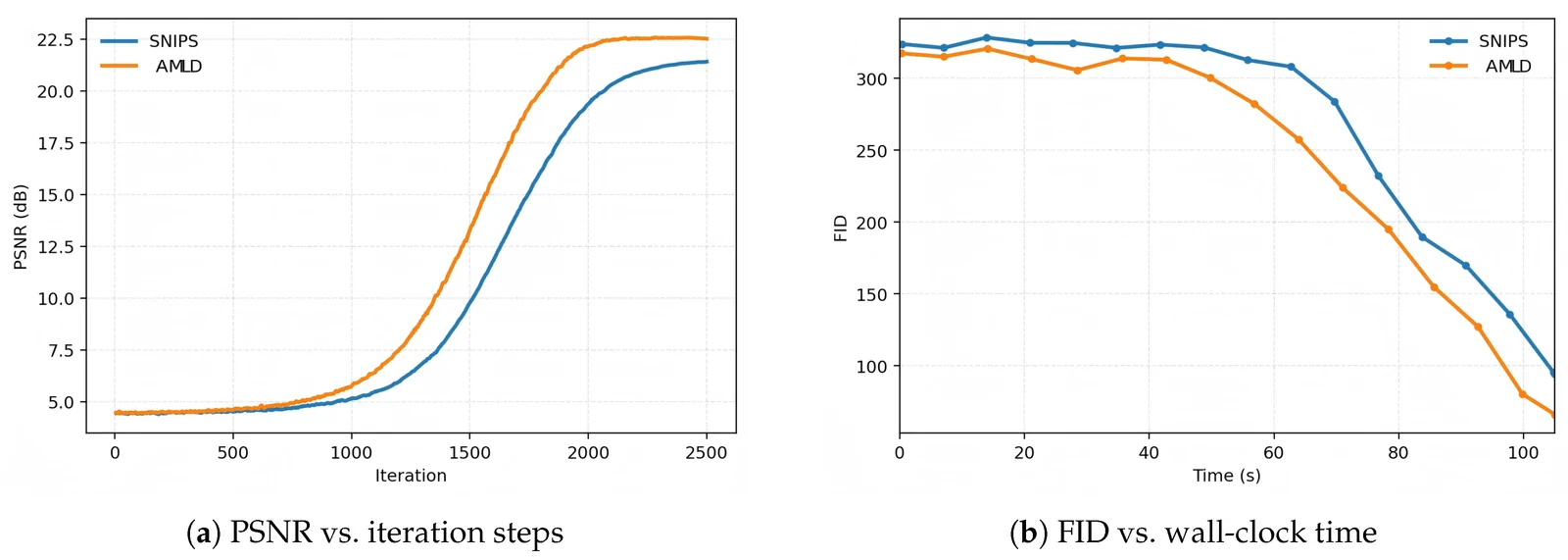
(**a**): PSNR versus iteration count. (**b**): FID versus wall-clock time. AMLD generally attains a target reconstruction quality using fewer sampling iterations than SNIPS, while maintaining a comparable overall runtime.

Figure 1 compares reconstruction quality as a function of iteration count and wall-clock time. AMLD typically reaches a given quality level using fewer sampling iterations than SNIPS. Although the momentum update introduces a small additional vector operation per iteration, the overall runtime remains comparable to SNIPS because score-network evaluation continues to dominate the computational budget.

From an implementation perspective, AMLD is easy to integrate into existing annealed posterior samplers. The additional state variable consists mainly of an auxiliary momentum vector, so the method acts as a lightweight drop-in replacement for the standard first-order correction kernel.

### 4.5. Visualization of the Momentum Schedule

**Figure 2:**
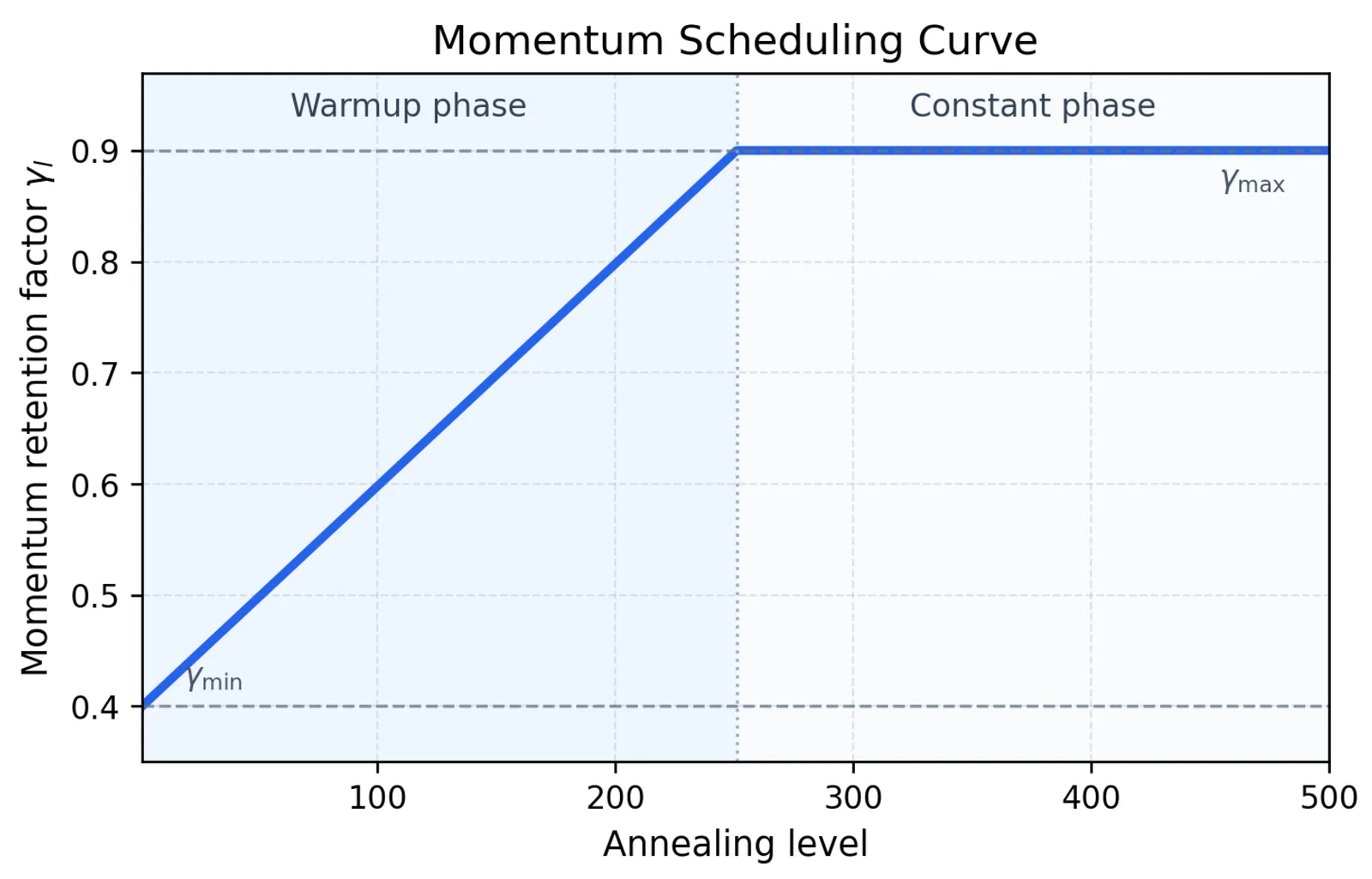
Evolution of the momentum retention factor $\gamma_{l}$ across annealing levels for the default AMLD configuration. During the warmup phase (first half of levels), $\gamma_{l}$ increases linearly from $\gamma_{\min}$ to $\gamma_{\max}$, and then remains constant for the remaining levels.

Figure 2 illustrates the evolution of the momentum retention factor $\gamma_{l}$ across annealing levels for the default AMLD configuration ($\gamma_{\min}=0.4$, $\gamma_{\max}=0.9$, warmup ratio r=0.5).

The piecewise linear schedule in Figure 2 reflects the design principle of AMLD: in early high-noise stages, a lower retention factor prevents momentum from accumulating excessively over the broad, rough energy surfaces typical of large $\sigma_{l}$, which helps maintain numerical stability. In later low-noise stages, where the posterior landscape sharpens, the full retention factor $\gamma_{\max}$ accelerates convergence toward fine posterior modes by preserving directional information across correction steps.

Because $\gamma_{l}$ is constant within each annealing level and only changes between levels, the schedule introduces negligible computational overhead while providing a coarse-to-fine adaptation of momentum persistence that matches the evolving geometry of the annealed posterior sequence.

### 4.6. Qualitative Analysis

**Figure A1:**
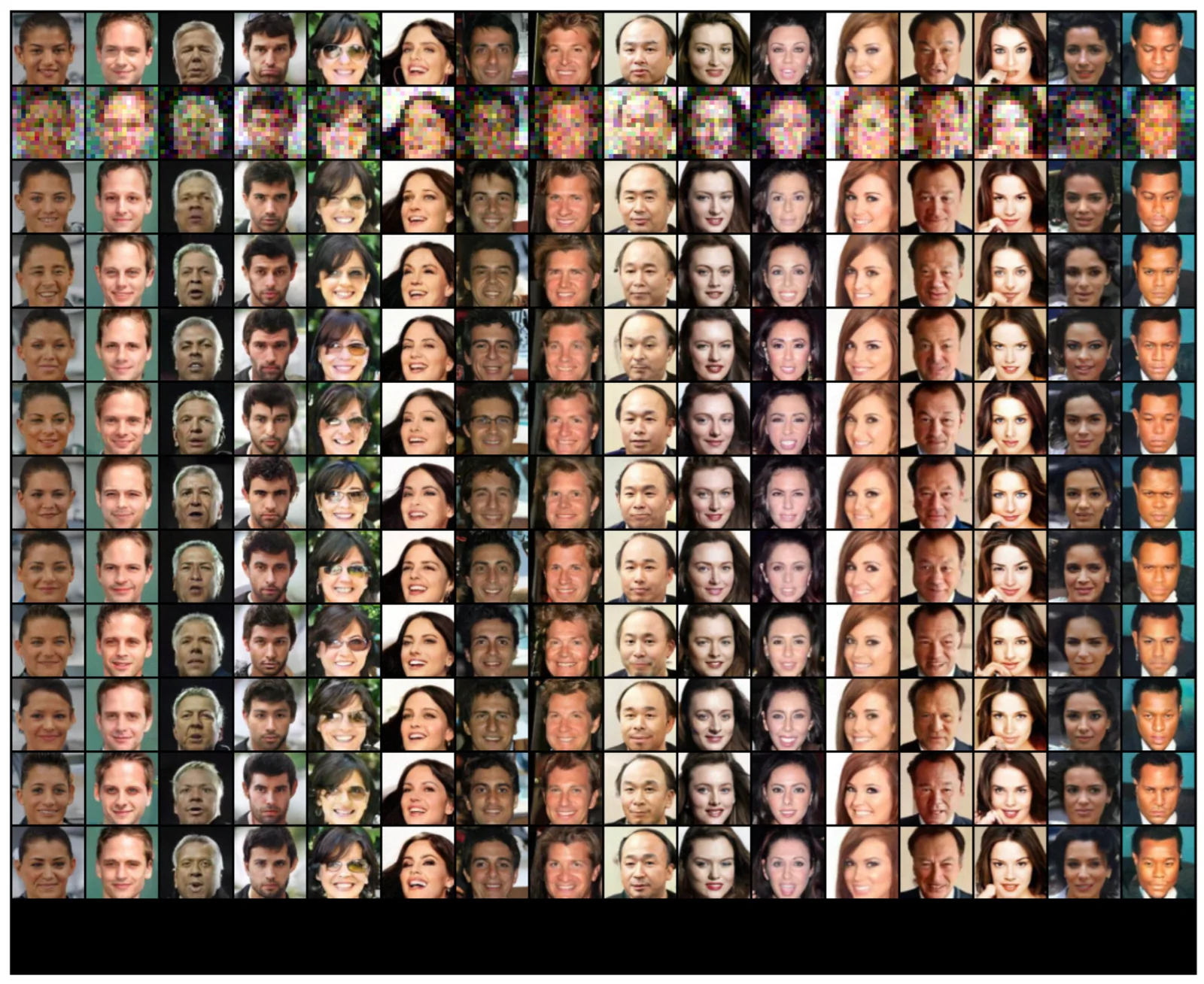
SR × 4 task: 10 representative examples of SNIPS reconstruction results. The last row shows per-pixel sample standard deviation maps.

**Figure A2:**
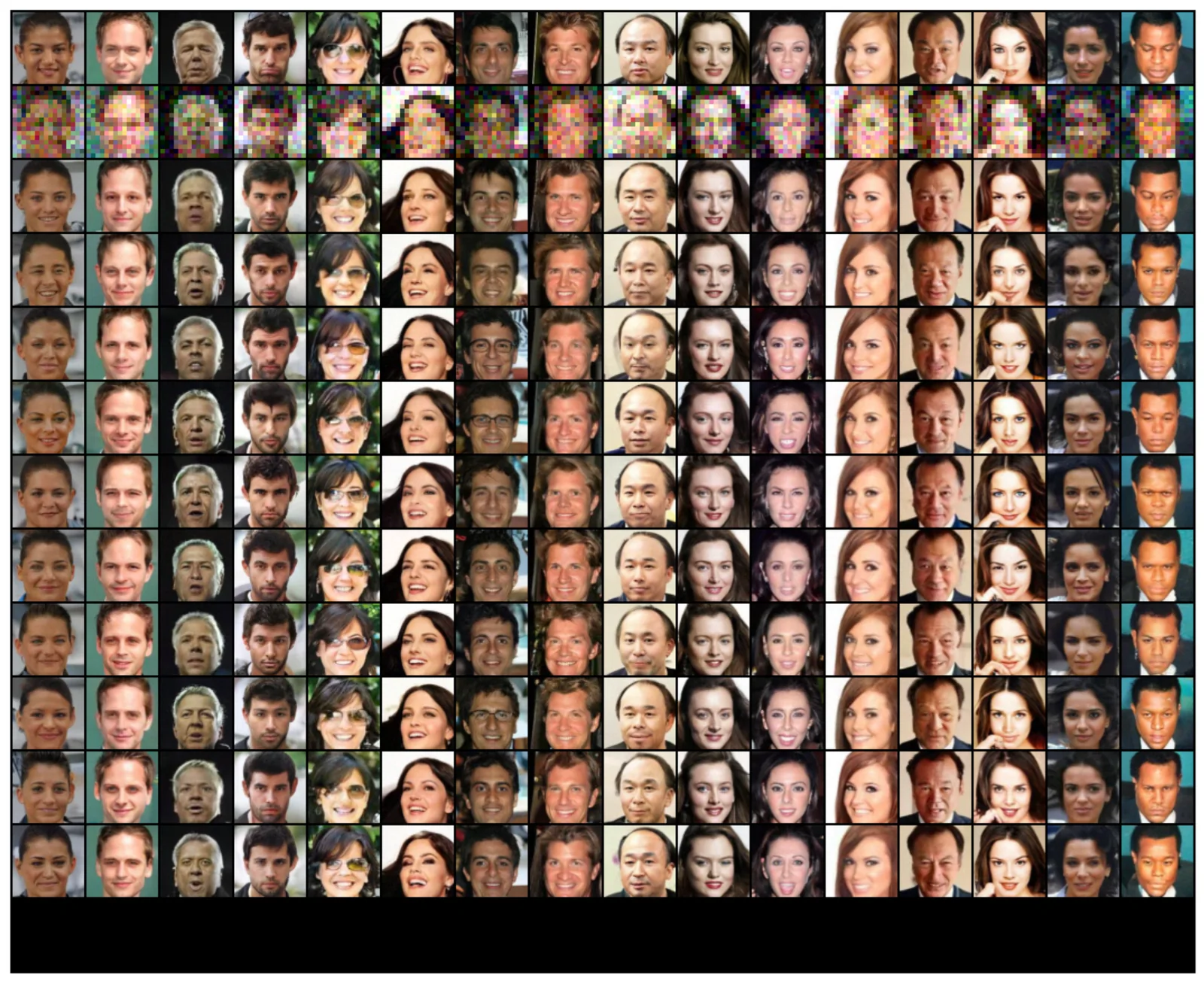
SR × 4 task: 10 representative examples of AMLD reconstruction results. The last row shows per-pixel sample standard deviation maps.

**Figure A3:**
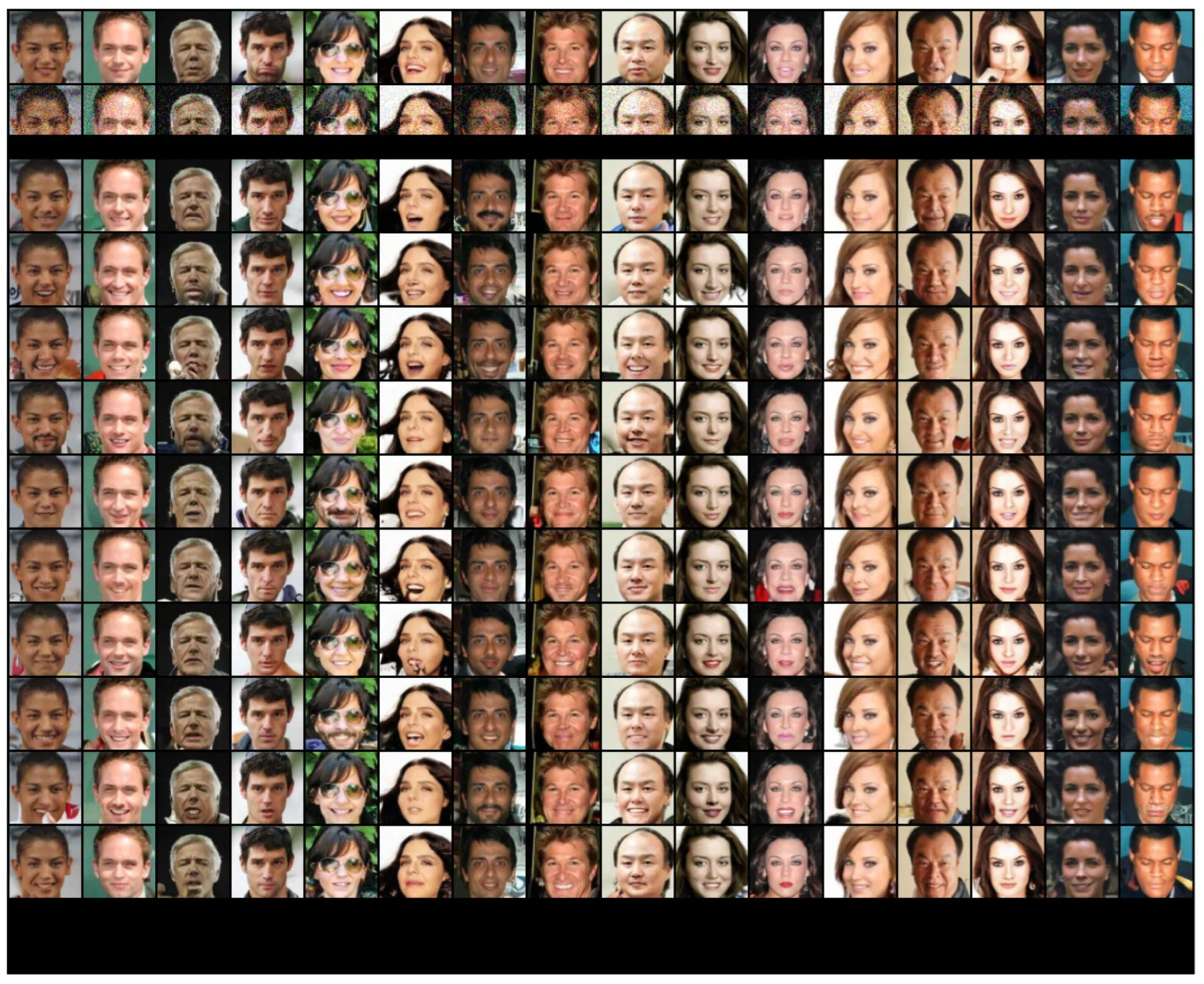
Inpainting task: 10 representative examples of SNIPS reconstruction results. The last row shows per-pixel sample standard deviation maps.

**Figure A4:**
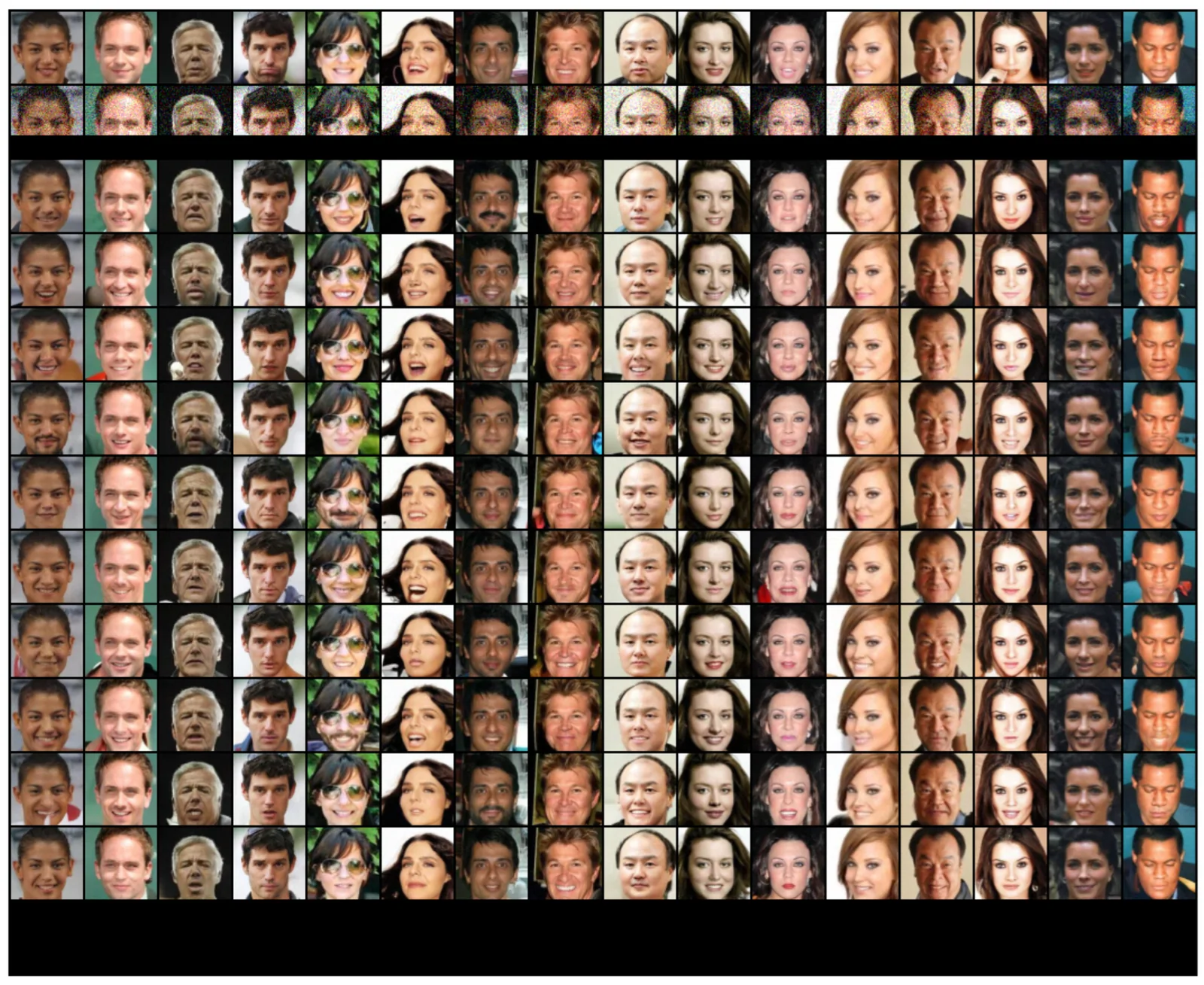
Inpainting task: 10 representative examples of AMLD reconstruction results. The last row shows per-pixel sample standard deviation maps.

**Figure A5:**
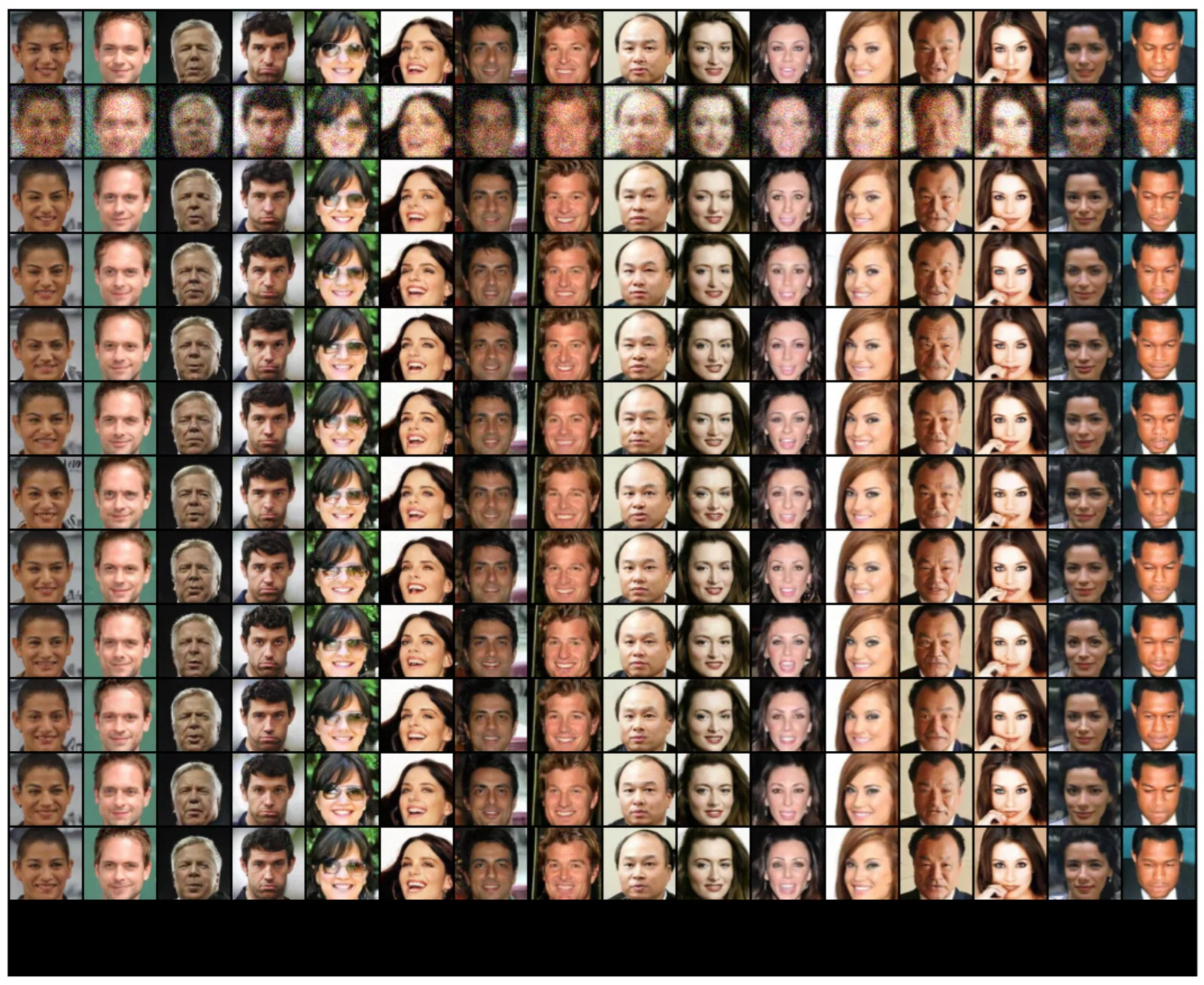
Gaussian deblurring task: 10 representative examples of SNIPS reconstruction results. The last row shows per-pixel sample standard deviation maps.

**Figure A6:**
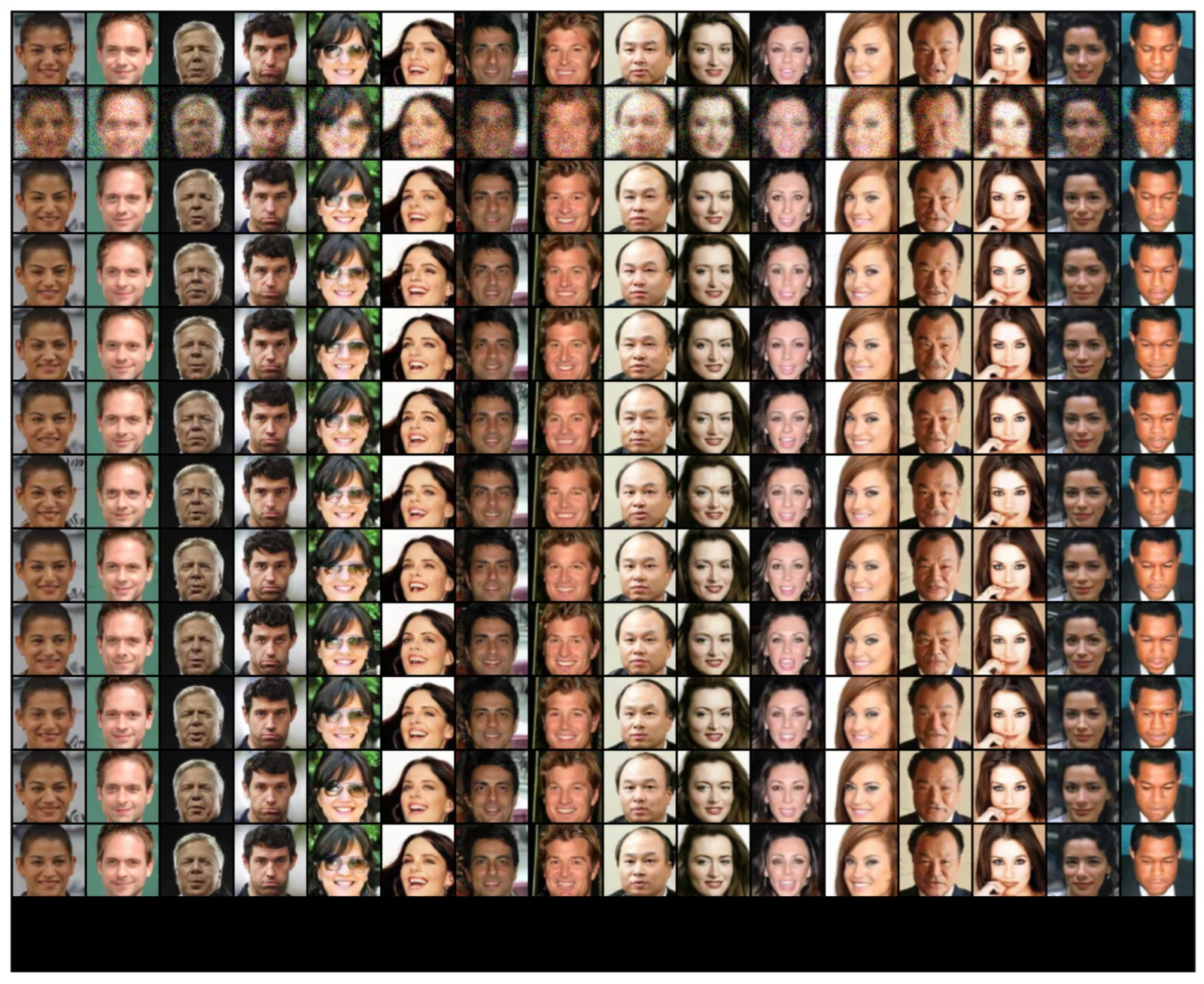
Gaussian deblurring task: 10 representative examples of AMLD reconstruction results. The last row shows per-pixel sample standard deviation maps.

Figure A1, Figure A2, Figure A3, Figure A4, Figure A5 and Figure A6 in Appendix A compare SNIPS and AMLD across all tasks. AMLD generally produces sharper edges, cleaner local textures, and more coherent reconstructions in regions with high-frequency structure. The per-pixel standard deviation maps shown in the last row of each figure also suggest more stable posterior refinement behavior, especially in visually ambiguous regions.

### 4.7. Ablation Study

To clarify the contribution of each component of AMLD, we conduct an ablation study on the Gaussian deblurring task in the nearly noiseless setting ($\sigma_{0}=0.0002$), using 8 representative validation images from CelebA.

Starting from the plain annealed Langevin sampler used in SNIPS (no momentum, first-order position update), we incrementally introduce the components of AMLD:

1. Fixed moderate momentum with a constant retention factor γ=0.5 across all annealing levels (no warmup schedule).
2. Fixed peak momentum with a constant retention factor $\gamma =\gamma_{\max}=0.9$ across all annealing levels (no warmup schedule).
3. Full AMLD with the annealing-aware momentum schedule ($\gamma_{\min}=0.4$, $\gamma_{\max}=0.9$, warmup ratio r=0.5) as defined in Equation (9).

All variants share the same score network, annealing schedule, step size, and evaluation protocol, so that performance differences can be attributed directly to the sampling update.

As shown in Table 4, reconstruction quality improves monotonically as AMLD components are introduced. The largest gain comes from adding momentum itself, which increases PSNR from 38.69 dB to 41.02 dB and substantially reduces LPIPS. This indicates that annealed posterior correction benefits substantially from a velocity variable that carries useful directional information across steps.

Increasing the momentum retention factor from moderate (γ=0.5) to peak ($\gamma =\gamma_{\max}=0.9$) yields a further improvement, raising PSNR from 41.02 dB to 41.87 dB while slightly reducing LPIPS. This suggests that higher momentum persistence is beneficial once the sampler can stably exploit directional information across the annealed trajectory.

Finally, adding the annealing-aware schedule changes the reconstruction metrics only marginally (41.87 dB to 41.89 dB). This is consistent with its intended role as a stability-oriented refinement rather than the primary source of empirical improvement: the warmup schedule mainly prevents early-stage instability when momentum is introduced abruptly, while the final asymptotic performance is determined primarily by the peak retention factor. Overall, the ablation study indicates that the main gains of AMLD arise from momentum itself and from operating at a sufficiently high retention factor, while the annealing-aware schedule provides an additional but modest stability benefit.

## 5. Conclusions

We presented Adaptive Momentum Langevin Dynamics (AMLD), a practical posterior sampling method for score-based inverse problems. AMLD replaces the conventional first-order Langevin correction with a momentum-based update equipped with an annealing-aware momentum retention schedule, while retaining the coordinate-wise step mechanism of SNIPS.

Methodologically, AMLD is motivated by underdamped Langevin dynamics on an extended state space, but is designed as a practical correction kernel for annealed posterior sampling rather than as an exact invariant discretization. This perspective is well aligned with score-based inverse problems, where posterior sampling itself is implemented through a finite sequence of stochastic refinement steps across noise levels.

Experimental results on Gaussian deblurring, image inpainting, and super-resolution demonstrate that AMLD achieves consistently competitive or superior reconstruction quality under both nearly noiseless and noisy conditions. The ablation study further shows that the primary empirical gains come from momentum itself and from operating at a high retention factor, while the annealing-aware schedule acts mainly as a lightweight stability refinement. In addition, AMLD is easy to implement and introduces only negligible overhead beyond score-network evaluation.

Overall, the results suggest that improving the correction dynamics—rather than only the prior model or annealing schedule—is a promising direction for advancing score-based posterior sampling in inverse problems. Future work may study stronger theoretical characterizations of momentum-based annealed samplers, extend AMLD to more challenging forward models, and explore its integration with larger and more advanced score-based generative priors.

## Figures and Tables

**Table 1:** Main notation used in Section 3.

Symbol	Meaning
$x\in R^{d}$	Unknown clean image (Bayesian model)
$y\in R^{m}$	Observed degraded measurement
$H\in R^{m\times d}$	Known linear degradation operator
n	Additive measurement noise
$\sigma_{n}$	Standard deviation of measurement noise
$\sigma_{l}$	Algorithmic annealing noise level at stage *l*
*L*	Number of annealing levels
*K*	Number of inner correction steps per level
$x_{t},\;m_{t}$	Continuous-time position and momentum (reference model)
$x_{k}^{\left(l\right)}$	Discrete position at annealing level *l*, inner step *k*
m	Momentum variable (initialized once; carried across levels)
$g_{k}^{\left(l\right)}=-\nabla U_{l}(x_{k}^{\left(l\right)})$	Posterior score at level *l*, step *k*
$s_{l}\in R^{d}$	Coordinate-wise step-size vector (inherited from SNIPS)
$s_{\theta }(\cdot ,\sigma_{l})$	Pre-trained score network at noise level $\sigma_{l}$
$U_{l}(\cdot )$	Annealed negative log-posterior energy (Equation (4))
γ	Scalar friction coefficient in reference dynamics
$\gamma_{\min},\;\gamma_{\max}$	Lower and upper bounds of the momentum retention factor
r∈(0,1]	Momentum warmup ratio across annealing levels

**Table 2:** Quantitative results on nearly noiseless images ($\sigma_{0}=0.0002$) from CelebA. Average PSNR [dB] ↑, LPIPS ↓, and FID ↓ for Gaussian deblurring, inpainting, and 4× super-resolution. Best results per metric are in **bold**.

Method	Deblurring (Gauss)			Inpainting			SR × 4		
	PSNR↑	LPIPS↓	FID↓	PSNR↑	LPIPS↓	FID↓	PSNR↑	LPIPS↓	FID↓
DDRM	38.88	0.0013	**23.93**	19.01	0.0412	94.86	25.43	0.0262	85.06
DPS	20.59	0.0282	167.77	11.77	0.358	267.16	13.90	0.0786	156.52
SNIPS	38.59	0.0007	34.13	18.94	0.0363	87.37	25.05	0.0266	88.48
**AMLD (Ours)**	**40.02**	**0.0006**	31.07	**20.47**	**0.0330**	**86.17**	**27.72**	**0.0258**	**84.71**

**Table 3:** Quantitative results on noisy images ($\sigma_{0}=0.1$) from CelebA. Average PSNR [dB] ↑, LPIPS ↓, and FID ↓ for Gaussian deblurring, inpainting, and 4× super-resolution. Best results per metric are in **bold**.

Method	Deblurring (Gauss)			Inpainting			SR × 4		
	PSNR↑	LPIPS↓	FID↓	PSNR↑	LPIPS↓	FID↓	PSNR↑	LPIPS↓	FID↓
DDRM	26.39	**0.0246**	**79.66**	18.43	0.056	**97.38**	22.03	**0.0536**	108.54
DPS	16.95	0.0536	114.32	11.76	0.229	211.31	12.66	0.114	117.67
SNIPS	25.41	0.0296	87.63	18.65	0.0447	105.22	21.47	0.0545	108.30
**AMLD (Ours)**	**26.48**	0.0294	83.40	**20.14**	**0.0420**	99.79	**22.32**	0.0540	**105.00**

**Table 4:** Ablation of AMLD components on CelebA Gaussian deblurring (nearly noiseless, $\sigma_{0}=0.0002$). Results are averaged over 8 validation images. PSNR [dB] ↑, LPIPS ↓. Best results are in **bold**.

Configuration	PSNR ↑	LPIPS ↓
C1: Plain Langevin (SNIPS baseline, no momentum)	38.69	0.0011
C2: +Fixed moderate momentum (γ=0.5, no schedule)	41.02	0.0005
C3: +Fixed peak momentum ($\gamma =\gamma_{\max}=0.9$, no schedule)	41.87	0.0004
C4: **Full AMLD** (annealing-aware momentum schedule)	**41.89**	**0.0004**

## Data Availability

The CelebA dataset is publicly available at https://mmlab.ie.cuhk.edu.hk/projects/CelebA.html (accessed on 17 July 2026). The score-network training code is based on the publicly available SNIPS repository [6]. Additional experimental details and configuration files are available from the corresponding author upon reasonable request.
